# Incidence, Risk Factors, and Time Trends for Bile Leakage After Cholecystectomy for Gallstone Disease—Results From a Population‐Based Cohort Study

**DOI:** 10.1002/wjs.70251

**Published:** 2026-02-19

**Authors:** Layla Mirzaei, Henrik Bergenfeldt, Stefan Öberg, Bodil Andersson

**Affiliations:** ^1^ Department of Clinical Sciences Lund Surgery Lund University and Helsingborg Hospital Helsingborg Sweden; ^2^ Department of Clinical Sciences Lund Surgery Lund University and Skåne University Hospital Lund Sweden

**Keywords:** bile leakage, cholecystectomy, risk factors, time‐trends

## Abstract

**Background:**

Bile leakage is a severe complication after cholecystectomy and is associated with an increased risk of morbidity and mortality. The aim of this study was to evaluate the incidence of bile leakage post‐cholecystectomy and to identify potential risk factors and their association with changes in the incidence of bile leakage over time.

**Methods:**

Demographic and perioperative data of all patients who underwent cholecystectomy in Sweden between 2006 and 2019 were retrieved from the Swedish Registry for Gallstone Surgery and Endoscopic Retrograde Cholangiopancreatography (GallRiks). Data on the occurrence of bile leakage within 30 days were recorded and risk factors were identified using uni‐ and multivariable logistic regression analyses.

**Results:**

Bile leakage occurred in 1738 of the 152,413 patients who underwent cholecystectomy, resulting in an overall incidence of 1.14%. The incidence was relatively consistent over the study period. ASA‐score II and III, emergent surgery, open cholecystectomy, conversion from laparoscopic to open technique, bleeding requiring intervention, not performing, or incomplete intraoperative cholangiography (IOC) were identified as risk factors for bile leakage. The proportion of ASA II and ASA III patients undergoing cholecystectomy increased over time (*p* < 0.001). There was also a significant increase in the proportions of emergent cholecystectomies from 27.9% to 43.6% (*p* < 0.001) and surgery for complicated gallstone disease from 35.4% to 52.5% (*p* < 0.001) during the study period.

**Conclusion:**

The incidence of bile leakage was relatively consistent over the study period despite an observed increase in the prevalence of identified risk factors of bile leakage.

## Graphical abstract

Bile leakage is a severe complication after cholecystectomy and is associated with an increased risk of morbidity and mortality. The aim of this study was to evaluate the incidence of bile leakage post‐cholecystectomy and to identify potential risk factors and their association with changes in the incidence of bile leakage over time. Demographic and perioperative data of all patients who underwent cholecystectomy in Sweden between 2006 and 2019 were retrieved from the Swedish Registry for Gallstone Surgery and Endoscopic Retrograde Cholangiopancreatography (GallRiks).

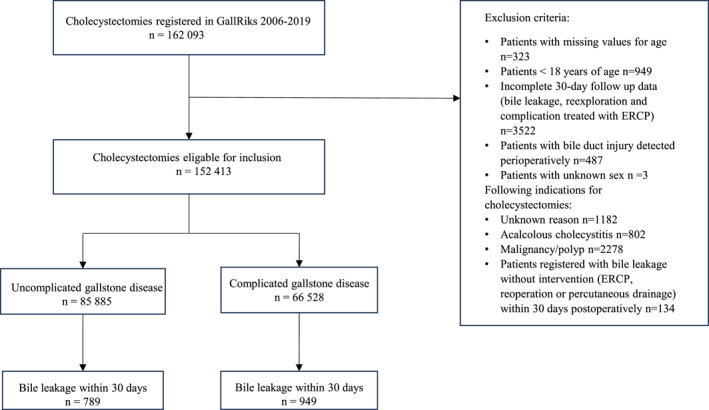

## 1 Introduction

Gallstone disease is a common and worldwide public health problem. Asymptomatic gallstones are present in up to 20% of the western populations, with an incidence that increases with age and is higher in females than males. Symptoms develop in about 20% of the population with gallstones [1, 2]. In the United Kingdom, gallstone disease accounts for the vast majority of acute admissions that require surgery [3].

Bile leakage is reported to occur in 0.4%–2.7% [4, 5, 6, 7, 8, 9, 10], making it the most common severe complication following cholecystectomy [4, 5, 6, 7]. Previous studies have reported female sex, emergent surgery, acute and chronic cholecystitis, and high American Society of Anesthesiologists (ASA) score to be risk factors for bile leakage [6, 7, 11, 12]. Although bile leakage is a rare complication, its association with increased morbidity and mortality constitutes a significant clinical concern [13].

Bile leakage often requires endoscopic and/or surgical interventions resulting in prolonged hospitalization with substantial health and economic effects [14, 15]. Bile leakage can arise from different types of injuries to the biliary tree. The most common sites of bile leakage include leakage from accessory ducts of the liver bed or the cystic duct stump. Bile leakage can also originate from major bile duct injuries (BDI), which include partial or complete transection of the common bile duct or the hepatic duct. They are uncommon, with a reported incidence of approximately 0.3% in patients undergoing cholecystectomies [16, 17].

The surgical techniques for treating gallstone disease have evolved considerably with the introduction of laparoscopic cholecystectomies in the 1980s. This approach has further evolved with the introduction of the critical view of safety (CVS), which is a standardized method for dissection and visualization of the biliary structures in order to minimize the risk for iatrogenic BDI [18]. Currently, the CVS is known and adopted increasingly by surgeons [19]. Furthermore, the patient population undergoing cholecystectomy has changed, with an increasing proportion of procedures performed in elderly patients who often have a substantial comorbidity burden [20, 21].

It is unknown if changes in surgical techniques, the patient population undergoing cholecystectomies, or changes in the proportion of complicated gallstone disease have affected the incidence of postoperative bile leakage. The primary aim of this study was to evaluate the incidence of bile leakage over time in patients undergoing cholecystectomies in Sweden. A secondary aim was to identify potential risk factors for bile leakage and assess if changes in the prevalence of these risk factors are associated with changes in the incidence of bile leakage.

## 2 Materials and Methods

### 2.1 Data Source

The Swedish Registry for Gallstone Surgery and Endoscopic Retrograde Cholangiopancreatography, ERCP (GallRiks) was established in May 2005. The database includes over 90% of all cholecystectomies performed in Sweden and it is continuously validated. Validations of GallRiks have demonstrated high accuracy and completeness of data [2, 22]. The registry contains information regarding perioperative data and outcomes for patients undergoing cholecystectomy and ERCP.

### 2.2 Study Design

All patients aged 18 years and older who underwent cholecystectomy for gallstone disease between January 1, 2006, and December 31, 2019, were included in this retrospective registry‐ and population‐based cohort study. Patients with incomplete 30‐day follow‐up with missing data regarding the occurrence of bile leakage, reoperations, or complications treated with ERCP were excluded from the study population. Additionally, patients with bile duct injury detected intraoperatively, unspecified patient sex, and patients undergoing surgery for acalculous cholecystitis, gallbladder malignancy, and/or polyps or cholecystectomy for unspecified reasons were also excluded.

In accordance with the definition of the registry, bile leakage was classified as a collection of intraabdominal bile within 30 days after the cholecystectomy requiring an intervention. Patients registered as having bile leakage without additional treatment, including ERCP, reoperation, or without percutaneous drainage intra‐ or postoperatively, were inconsistent with the definition and were therefore excluded from the study population. Emergent procedures were defined as cholecystectomies performed in patients during acute admissions regardless of the time from admission. Bleeding requiring intervention was defined as blood transfusion or the need for conversion to open surgery.

Patients in whom biliary colic was the indication for surgery were defined as having uncomplicated gallstone disease, whereas patients with acute cholecystitis, acute pancreatitis, previous cholecystitis, previous pancreatitis, and obstructive jaundice due to choledocholithiasis were defined as having a complicated gallstone disease. Patients were categorized into five relatively equally sized age groups (18–29, 30–44, 45–59, 60–74 and ≥ 75 years). This study was carried out according to the Strengthening the Reporting of Observational Studies in Epidemiology (STROBE) reporting guidelines [23].

### 2.3 Statistical Analysis

Categorical variables were expressed as numbers and percentages (%), and continuous variables were expressed as median and 25th−75th percentile as they had nonnormally distribution patterns. Consequently, comparisons of continuous variables between two groups were made using the Mann–Whitney *U*‐test. Comparisons of categorical variables were made using the chi‐square test. Univariable binary logistic regression analyses and extraction of odds ratios (OR) and their 95% confidence intervals (CIs) were performed for the identification and assessment of risk factors for bile leakage. Multivariable logistic regression analyses were used to evaluate associations between multiple factors for bile leakage. Variables considered clinically important and potentially affected outcomes were entered simultaneously for adjustments in the regression models. The factors adjusted for included patient age and sex, ASA‐classification, emergent surgery, the surgical approach, intraoperative cholangiography (IOC), the occurrence of bleeding that required intervention, and the study period categorized in time intervals of 3 years.

Linear regression analyses were used to analyze potential differences in the proportion of identified risk factors and the occurrence of bile leakage over time. A sensitivity analysis using Poisson regression was conducted to validate the results of the linear regression analysis. All tests were two‐sided, and a *p*‐value < 0.05 was considered significant. Relative risk ratio was used to measure the association between the exposure and outcome.

Statistical analyses were performed using STATA/SE version18 (StataCorp LLC).

## 3 Results

### 3.1 Description of the Study Population

**FIGURE 1:**
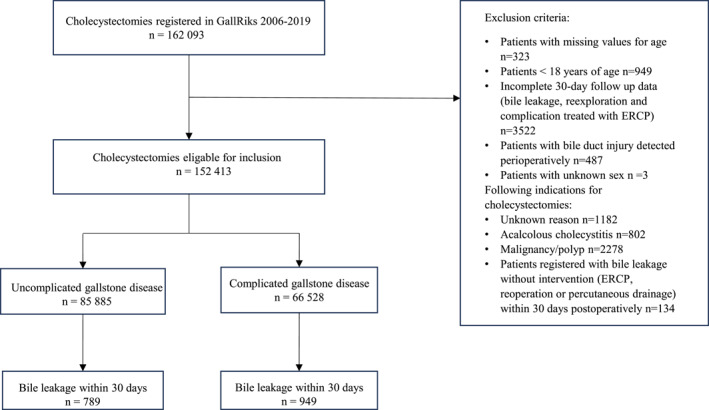
Flowchart of the study cohort.

A total of 162,093 patients who underwent cholecystectomies in Sweden between 2006 and 2019 were identified from the national registry. After excluding 9680 patients according to the exclusion criteria, the study population consisted of 152,413 patients (Figure 1). Bile leakage was detected postoperatively in 1738 patients, resulting in an overall incidence of 1.1%. Patients with bile leakage were significantly older than patients without bile leakage (Table 1). Almost two‐thirds of the patients were females, and a majority of the patients had an ASA score of II‐III. Bile leakage was significantly more common in females compared with males. The majority of the patients were operated for uncomplicated gallstone disease. Complicated gallstone disease was registered in 66,528 patients in whom 79,949 types of complicated gallstone disease were recorded, indicating that many patients had more than one type of complicated gallstone disease. Acute cholecystitis was the most common type of complicated disease, accounting for 48% of these cases. Bile leakage was significantly more common in patients with acute or previous cholecystitis, acute pancreatitis, and obstructive jaundice. Emergent cholecystectomies were performed in 34% of the patients of whom 78% had a complicated gallstone disease. Bile leakage within 30 days was significantly more common after emergent procedures, with a relative risk ratio of 1.6 times higher than that of patients who underwent elective procedures. Furthermore, bile leakage was significantly more common in patients with perioperative bleeding that required interventions and in patients in whom cholangiography was incomplete or not performed at all (Table 2).

**TABLE 1:** Overall results including demographics and clinical characteristics in patients with and without bile leakage.

	Overall (*n* = 152,413)	Bile leakage (*n* = 1738)	No bile leakage (*n* = 150,675)	*p*‐value
Median age (years)	51 (38–64)	57 (43–68)	51 (38–64)	< 0.001
Age group (years)				
18–29	16,426 (10.8)	113 (0.7)	16,313 (99.3)	< 0.001
30–44	40,045 (26.3)	355 (0.9)	39,690 (99.1)	
45–59	45,210 (29.7)	506 (1.1)	44,704 (98.9)	
60–74	39,159 (25.7)	561 (1.4)	38,598 (98.6)	
> 75	11,573 (7.6)	203 (1.8)	11,370 (98.2)	
Sex				
Female	100,918 (66.2)	1090 (1.1)	99,828 (98.9)	0.002
Male	51,495 (33.8)	648 (1.3)	50,847 (98.7)	
ASA‐score				
I	72,229 (47.4)	634 (0.9)	71,595 (99.1)	< 0.001
II	66,673 (43.7)	837 (1.3)	65,836 (98.7)	
III	13,048 (8.6)	252 (1.9)	12,796 (98.1)	
IV–V	463 (0.3)	15 (3.2)	448 (96.8)	
Severity of the disease				
Uncomplicated gallstone disease	85,885 (56.4)	789 (0.9)	85,096 (99.1)	< 0.001
Complicated gallstone disease	66,528 (43.6)	949 (1.4)	65,579 (98.6)	
Type of complicated gallstone disease				
Acute cholecystitis	31,668 (20.7)	545 (1.7)	31,123 (98.3)	< 0.001
Acute pancreatitis	5761 (3.8)	45 (0.8)	5716 (99.2)	0.009
Previous cholecystitis	18,535 (12.2)	327 (1.8)	18,208 (98.2)	< 0.001
Previous pancreatitis	9641 (6.3)	124 (1.3)	9517 (98.7)	0.163
Obstructive jaundice	14,344 (9.4)	223 (1.6)	14,121 (98.4)	< 0.001
Timing				
Elective	100,101 (65.7)	951 (0.9)	99,150 (99.1)	< 0.001
Emergent	52,312 (34.3)	787 (1.5)	51,525 (98.5)	

*Note:* Values expressed as numbers (%) or medians (i.q.r).

Abbreviation: ASA, American Society of Anestesiologists.

**TABLE 2:** Intraoperative data in patients with and without bile leakage.

	Total (*n* = 152,413)	Bile leakage (*n* = 1738)	No bile leakage (*n* = 150,675)	*p*‐value
Surgical approach				
Laparoscopic cholecystectomy	133,927 (87.8)	1157 (0.9)	132,770 (99.1)	< 0.001
Converted to open cholecystectomy	9131 (6.0)	339 (3.7)	8792 (96.3)	
Open cholecystectomy	9199 (6.1)	237 (2.6)	8962 (97.4)	
Subtotal cholecystectomy	156 (0.1)	5 (3.2)	151 (96.8)	
Cholangiography				
Complete IOC	131,789 (86.5)	1286 (1.0)	130,503 (99.0)	< 0.001
No or incomplete IOC	20,624 (13.5)	452 (2.2)	20,172 (97.8)	
Bleeding				
Yes	1073 (0.7)	43 (4.0)	1030 (96.0)	< 0.001
No	151,340 (99.3)	1695 (1.1)	149,645 (98.9)	

*Note:* Values expressed as numbers (%).

The origin of bile leakage was the cystic duct in 44%, whereas accessory ducts from the liver bed were the site of leakage in 21% of the patients. In 27% of the patients, the origin was unknown, and 8% had other origins of leakage of which 4% were due to major bile duct injuries. All data were complete without missing values apart from 6 patients missing data on the origin of bile leakage.

### 3.2 Potential Risk Factors for Bile Leakage

In univariable logistic regression analyses, there were significant associations between the occurrence of bile leakage and increasing patient age, male sex, ASA‐class II, III and IV, surgery for complicated gallstone disease, emergent surgery, and bleeding requiring intervention (Table 3). Furthermore, conversion from laparoscopic to open surgery and the performance of open and subtotal cholecystectomy were significantly associated with the occurrence of bile leakage. Refrainment from performing IOC or an incomplete IOC was also associated with increased risks of bile leakage. There was a significant difference in the incidence of bile leakage in patients within different age groups. In both univariable and the multivariable analyses, there was an increased risk of bile leak with increasing age. Other factors significantly associated with bile leakage in the multivariable analysis included ASA‐score II and III, emergent surgery, conversion to open cholecystectomy, primary open cholecystectomy, bleeding, and incomplete or refrainment from IOC. There was no significant difference in the incidence of bile leakage after cholecystectomies performed during the different time periods of this study.

**TABLE 3:** Logistic regression analyses showing the association between potential risk factors and the occurrence of bile leakage.

	Univariable analysis			Multivariable analysis		
Variables	OR	95% CI	*p*‐value	OR	95% CI	*p*‐value
Age (years)						
18–29	Ref			Ref		
30–44	1.29	1.04–1.60	0.018	1.24	1.00–1.54	0.046
45–59	1.63	1.33–2.01	< 0.001	1.39	1.13–1.72	0.002
60–74	2.10	1.71–2.57	< 0.001	1.50	1.21–1.85	< 0.001
> 75	2.58	2.05–3.25	< 0.001	1.37	1.07–1.76	0.013
Sex						
Female	Ref			Ref		
Male	1.17	1.06–1.29	0.002	0.91	0.83–1.01	0.080
ASA‐score						
I	Ref			Ref		
II	1.44	1.29–1.59	< 0.001	1.16	1.04–1.30	0.008
III	2.22	1.92–2.58	< 0.001	1.47	1.25–1.74	< 0.001
IV–V	3.78	2.25–6.36	< 0.001	1.69	0.98–2.89	0.057
Timing						
Elective	Ref			Ref		
Emergent	1.59	1.45–1.75	< 0.001	1.21	1.10–1.34	< 0.001
Surgical approach						
Laparoscopic cholecystectomy	Ref			Ref		
Converted to open cholecystectomy	4.42	3. 91–5.00	< 0.001	3.44	3.01–3.93	< 0.001
Open cholecystectomy	3.03	2.63–3.49	< 0.001	2.50	2.13–2.92	< 0.001
Subtotal cholecystectomy	3.80	1.56–9.28	0.003	2.12	0.86–5.22	0.100
Cholangiography						
Complete IOC	Ref			Ref		
No or incomplete IOC	2.27	2.04–2.53	< 0.001	1.93	1.73–2.16	< 0.001
Bleeding						
No	Ref			Ref		
Yes	3.69	2.71–5.02	< 0.001	1.92	1.40–2.64	< 0.001
Time intervals						
2006–2008	1.06	0.90–1.25	0.497	0.87	0.73–1.03	0.104
2009–2011	1.02	0.87–1.19	0.785	0.89	0.76–1.04	0.158
2012–2014	1.13	0.97–1.31	0.111	1.08	0.93–1.26	0.294
2015–2017	0.92	0.79–1.08	0.340	0.93	0.79–1.08	0.345
2018–2019	Ref					

### 3.3 Risk Factors for Bile Leakage Over Time

**FIGURE 2:**
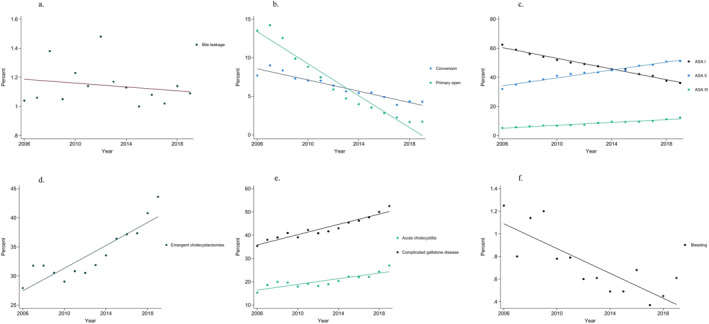
(a–f) Time trends for the incidence of bile leakage and identified risk factors. (a) Incidence of bile leakage over time. (b) Proportion of cholecystectomies converted from laparoscopic to open and primary open. (c) Proportion ASA‐score I–III. (d) Proportion emergent cholecystectomies. (e) Proportion complicated gallstone disease and acute cholecystitis. (f) Proportion bleeding requiring intervention.

Using linear regression analysis, there was no significant difference in the incidence of bile leakage over time (*p* = 0.715) (Figure 2a) but a significant increase in the proportion of laparoscopic surgery from 79% to 94% (*p* < 0.001), corresponding to a 1.4% yearly increase during the study period. During the same time period, there was a decrease in cholecystectomies converted from laparoscopic to open approach (*p* < 0.001) and primary open cholecystectomies (*p* < 0.001) (Figure 2b). Furthermore, the proportion of ASA I patients decreased from 62% to 36% (*p* < 0.001), corresponding to a decrease of 1.8% per year, whereas the proportion of ASA II (*p* < 0.001) and ASA III patients increased (*p* < 0.001) (Figure 2c). The proportion of patients in the age group 60–74 years increased significantly from 24% to 27% (*p* < 0.001). Concurrently, the proportion of emergent cholecystectomies increased significantly from 28% to 44% (*p* < 0.001), corresponding to a yearly increase of 1% (Figure 2d).

The incidence of surgery for complicated gallstone disease increased significantly over time, with 1.1% per year from 35% to 52% (*p* < 0.001). In patients with complicated gallstone disease, the proportion of acute cholecystitis increased from 15% to 27% (*p* < 0.001) (Figure 2e). The proportion of perioperative bleeding that required intervention decreased from 1.3% to 0.6% during the study period (*p* < 0.001) (Figure 2f). Furthermore, there was a decrease in the proportion of patients with incomplete IOC or in whom there was made no attempt to perform IOC from 17% to 11% (*p* < 0.001).

## 4 Discussion

This nationwide registry‐based study exploring the incidence of post cholecystectomy bile leakage and its risk factors over time found that the incidence of bile leakage was relatively consistent during the 14‐year study period. Concurrently, the proportion of the identified risk factors such as emergent surgery and high ASA‐score increased significantly.

This is, to our knowledge, the largest study conducted on bile leakage after cholecystectomies and the only study assessing the incidence of bile leakage and its associated risk factors over time. The observed incidence of bile leakage was 1.14% similar to previous studies that reported incidences ranging from 0.38% to 2.7% [4, 5, 6, 7, 8, 9, 10]. Vecchio and coworkers reported an incidence of 0.38% in a large cohort who underwent laparoscopic cholecystectomies between 1989 and 1995 [8]. However, as information regarding the occurrence of acute cholecystitis or other types of complicated gallstone disease was lacking in most patients, it is possible that the study was conducted mainly on a low‐risk population, potentially explaining the relatively low incidence of bile leakage. In a similar study consisting of 264 laparoscopic cholecystectomies performed between 1990 and 1991, bile leakage was detected in 2.7% of the patients [10]. That study focused on postoperative imaging for detecting bile leakage and also lacked information regarding the occurrence of complicated gallstone disease. However, all patients with bile leakage were considered clinically significant and in need of further treatment with either surgery or ERCP. Other more recent studies on post‐cholecystectomy bile leakage, which are smaller in size, report an incidence of bile leakage similar to that of the current study [6, 12, 13, 14, 16].

The proportion of emergent cholecystectomies increased significantly and was almost two times higher at the end of the study period. Patients who underwent emergent procedures had an increased risk of bile leakage, with a relative risk ratio of 1.6 compared with that of patients having elective procedures. Emergent cholecystectomies have also previously been described as a significant risk factor for bile leakage. A study by Nassar et al. reported a two‐fold higher rate of bile leakage after emergency cholecystectomies [12]. Similarly, Shaik et al. described an almost threefold higher rate of bile leakage from the cystic duct in patients undergoing emergent laparoscopic cholecystectomy compared with elective procedures [24]. Even though both studies are considerably smaller, the incidence of bile leakage for emergent procedures was similar to the observations in the present study.

As a result of the publication of new studies and updated guidelines during the study period, the timing of cholecystectomy in patients with cholecystitis and mild pancreatitis may have changed [1, 25, 26, 27, 28, 29]. These studies have reported benefits of early cholecystectomy also in severe cases, high risk patients and elderly. This may have resulted in more same‐admission cholecystectomies, defined as emergent procedures in this study. However, these potential changes in clinical management do not seem to have affected the rates of bile leakage.

The present study identified several other factors associated with bile leakage after cholecystectomy. Patients with ASA‐score II and III, perioperative bleeding, open cholecystectomy, or laparoscopic cholecystectomy converted to an open approach and not performing or incomplete cholangiography were also significant risk factors for bile leakage. Apart from a previous study based on the GallRiks database, which also found high ASA‐scores associated with an increased risk of bile leakage, the other risk factors have not been reported earlier. However, a high ASA‐score has been reported as a risk factor for mortality in a study of 216 patients with post‐cholecystectomy bile leakage [13]. In the present study, there was an increase in the proportion of elderly patients and patients with a high comorbidity burden over time, suggested by decreasing proportions of patients with ASA‐score I and increasing proportions of patients with ASA‐scores II and III. Consequently, the proportion of patients with high surgical risk increased over time.

There were significant changes in the surgical management of gallstone disease during the study period. The proportion of operations completed laparoscopically and procedures with complete cholangiography increased significantly, whereas perioperative bleeding requiring intervention decreased significantly during the study period. The finding that the performance of intraoperative cholangiography was associated with a significantly reduced risk of bile leakage is interesting as it would suggest that implementing routine cholangiography could theoretically reduce the incidence of bile leakage at centers currently not employing this strategy. However, as the current study is based on a retrospective review, the level of evidence may not be sufficient to recommend a change in clinical practice. The association between incomplete or omitted cholangiography and bile leakage could also be due to technical difficulties in completing a cholangiography in patients with very advanced cholecystitis. On the other hand, in procedures technically difficult, it may be even more important to perform cholangiography to avoid bile duct injuries and bile leakage [30]. Other procedural factors associated with a decreased risk of bile leakage included laparoscopic surgery and operations without significant perioperative bleeding, both of which increased during the study period. The relatively consistent incidence of bile leakage despite increasing proportions of patients with high ASA‐scores, complicated gallstone disease, in combination with decreasing proportions of perioperative bleeding and a higher proportion of procedures completed laparoscopically, suggests that the surgical technique improved over time. Furthermore, during the course of the study, there has been considerable advancements in laparoscopic instruments with higher quality cameras allowing the surgeons to perform more precise dissection with minimal bleeding. Also, there has been improvements in the accessibility and the quality of simulators for surgical training which may also have had beneficial effects on surgical technique.

The findings in this study provides important knowledge for the identification of patients with increased risk for bile leakage. To reduce the risk for bile leakage in high‐risk patients, several intraoperatively strategies may be implemented. These strategies may include adequate performance of CVS and IOC, proper surgical technique with avoidance of bleeding, intraoperative attention to identify aberrant bile ducts, and appropriate closure of the cystic duct.

### 4.1 Strengths and Limitations

There are several strengths but also some limitations in the present study. This type of large registry‐based studies has many advantages over previous single center series, as it provides information regarding the management and the results of surgery for gallstone disease in larger populations and at multiple centers, whereas single‐center studies reflect results from fewer surgeons and therefore lack in generalizability. Another strength is the homogeneity of the study population, which only included patients with clinically significant bile leakage that required intervention and further excluded patients with intraoperative bile duct injury. As there is no widely accepted definition of bile leakage, the definitions between studies may vary, affecting the comparability of study results. Limitations are similar to other nonrandomized registry studies, such as unidentified confounders, the unavailability of specific variables, and erroneous reporting.

Due to missing values in the data reported to the registry, variables not included in the assessment of risk factors for bile leakage included BMI, smoking habits, and the experience of the surgeons performing the cholecystectomy. Although it would have been desirable to include BMI in the assessments, previous studies have described laparoscopic cholecystectomy to be safe and effective in patients with morbid obesity, and high BMI has not been shown to be associated with poor outcomes [31, 32]. Previous studies show contradictory results regarding the impact of surgical experience and complications after cholecystectomy [33, 34, 35]. Two studies found no association between surgeon's volume and outcome. In another study, patients operated by low‐volume surgeons had significantly more surgical complications, including bile duct injuries in elective procedures, but no such difference was observed for emergent procedures.

## 5 Conclusion

In conclusion, this study showed that the incidence of bile leakage was relatively consistent during the study period despite a significant increase in the prevalence of identified risk factors for bile leakage. The reason for this is unclear but may be explained by improved surgical technique during the study period.

## Author Contributions

**Layla Mirzaei:** conceptualization, investigation, funding acquisition, writing – original draft, methodology, validation, visualization, writing – review and editing, software, formal analysis, project administration, data curation, resources. **Henrik Bergenfeldt:** conceptualization, investigation, methodology, validation, visualization, writing – review and editing, software, supervision, formal analysis, project administration, data curation. **Stefan Öberg:** conceptualization, funding acquisition, writing – review and editing, visualization, validation, methodology, formal analysis, supervision, resources, investigation. **Bodil Andersson:** conceptualization, funding acquisition, writing – review and editing, visualization, validation, methodology, formal analysis, supervision, resources, investigation.

## Funding

This research work was supported by grants from Stig and Ragna Gorthon Foundation for Medical Research, the Thelma Zoéga Foundation, Government Grant for Clinical Research (http://www.skane.se/fou/alf), and Region Skane Research Foundation.

## Disclosure

This study was previously presented as an abstract at the 16th Biennial Congress of the European‐African Hepato‐Pancreato‐Biliary Association, 10–12 June 2025 in Dublin, Ireland.

## Ethics Statement

The current study was approved by the Regional Research Ethics Committee in Stockholm, Sweden (Dnr 2021–01783).

## Consent

Patients have to be informed regarding registration in the registry at the time of surgery. However, an informed consent is not needed. Thus, almost all patients are registered but patients can apply to be removed from the registry.

## Conflicts of Interest

The authors declare no conflicts of interest.

## Data Availability

The data that support the findings of this study are available from the corresponding author upon reasonable request.
